# The Essence of Mental Training

**Published:** 1907-08-17

**Authors:** 


### Nursing Administration.

### THE ESSENCE OF MENTAL TRAINING.

The mental specialist, the asylum superintendent, is usually found looking askance at the hospital trained nurse when it is a question of entrusting his cases to her care. It is not that he has not a deep sense of her many talents. It is not that he does not, from many points of view, entirely concur with the system under which she has been trained; but he has found by experience that she does not suit his purpose. He prefers for the superintendence of his wards, for the conduct of his cases, a woman who, though she may be lacking in the knowledge only to be thoroughly acquired in the general hospital, possesses the root of the matter as regards mental patients. It is interesting to consider in what this vital quality consists, which may be imparted to a comparatively uneducated woman and be wanting in nurses deeply versed in the science and art of treating the sick.

The crux of the situation as regards the care of the insane is the conviction only to be gained by long familiarity with mental disease, of the power of the sane over the unbalanced mind. The sane person is one in harmony with prevailing conditions. The insane are at issue with their environment. Therein lies their weakness, and the vantage ground which they afford to their attendants. It is not a distinction of degree as regards mental or physical characteristics. The insane are very often, indeed, far cleverer, far stronger, far better educated than those who are called on to control their movements. But as regards the affairs of life they stand on a different plane. And the power of the attendant lies in his recognition of this fact. He governs by a sense of conscious superiority, having his own limited faculties well in hand, and able to calculate securely on the fact that the faculties against which he may have to contend in those under his charge are "unbridled," do not obey their master, and do not count. It is the victory achieved by the general of a small and well disciplined army keeping in check a great nation divided by factions and owning no leader.

Now this sense of being on a different plane to the mentally diseased is a sense which can only be acquired in its fullest extent by those who apply themselves to it young. It is probably never consciously taught. It grows in the mind, strengthened by frequent exercise, and by long association with those in whom it is second nature. It eliminates all fear even in the presence of the most violent outbreaks. For the same want of control which leads to violence offers the watchful attendant her opportunity, and she has been trained to take full advantage of such opportunities. The idea of arguing with a patient would never occur to a nurse trained to this perception of her own inherent unlikeness to her charge. She would never dream that the arguments which would appeal to her and to her kind would be of avail with this different order of beings. Nor does the exertion of force commend itself to her. She has learned the impotence of force by frequent object lessons in the behaviour of the insane themselves, and she would scorn to use her strength against creatures whom she has grown to regard as helpless, however physically gifted. Pity she will feel, affection she may often cultivate for those under her care, but to be rude, or angry, or impatient, as she might be to her own equals, lies outside the code of conduct towards those on the lower plane. Instead there is the reserve force, the force which sanity lends. Such very briefly is the attitude which goes to make up the essential part of the trained mental nurse. It is absolutely different, it will be perceived, to the attitude which the hospital nurse assumes towards her patients. The important lesson for the hospital nurse is the lesson that she and her patients are of the same flesh and blood. She is to regard them as units in the great sum of suffering humanity, and must before all else keep herself from the snare of setting them in a different category as mere cases. She must put herself in their place, and learn to see with their eyes, and hear with their ears. Her success as a private nurse will unquestionably depend on her ability to approach her patients on their own level.

We have finally to remember that when mind and body both are affected, the mind is the more important factor to be considered. The medical attendant prefers in such cases that body rather than mind shall go short if both cannot be equally well tended. Better that the consumptive suffering from melancholia should be shielded from needless irritation and kept mentally well in hand, even if his temperature charts are not particularly well kept. And little will the surgical skill of the trained nurse avail the patient whom she has permitted in an unguarded moment to tear the bandages from his wound. Moreover, there is the ever-recurrent danger of suicide to be borne in mind.

There does not appear to be much prospect of mental nurses in any great numbers supplementing their mental training by a course of hospital experience. The best outlook for medical and surgical nursing in asylums is improvement from within. Wide experience in the treatment of disease is not to be looked for in asylums, nor much practice in the routine work of the hospital ward. But this makes it all the more important that the small amount of experience and practice available for probationers shall be presented under good conditions. The principles of asepticism cannot properly be imparted in buildings constructed to set them at defiance. Much has been done of late years by the addition of well-equipped infirmary wards to render possible improved methods of nursing. Much is likely to be done in the immediate future through the more scientific treatment of lunacy as a disease susceptible of remedy at the hands of physician or surgeon. Only in proportion as asylums themselves come into line among the best institutions of the day for the treatment of disease will those trained within their walls be raised in status. Already be it said with hearty appreciation, the attendants in certain asylums, where training of a really efficient kind is given, will bear comparison with the highest products of the general hospital training schools.

